# Fungal Bioremediation of the β-Lactam Antibiotic Ampicillin under Laccase-Induced Conditions

**DOI:** 10.3390/antibiotics13050407

**Published:** 2024-04-29

**Authors:** Bouthaina Ghariani, Abdulrahman H. Alessa, Imen Ben Atitallah, Ibtihel Louati, Ahmad A. Alsaigh, Tahar Mechichi, Héla Zouari-Mechichi

**Affiliations:** 1 Laboratory of Biochemistry and Enzyme Engineering of Lipases, National School of Engineers of Sfax, University of Sfax, BP 1173, Sfax 3038, Tunisia; bouthaina.ghar@live.fr (B.G.); benatitallahimen@gmail.com (I.B.A.); ibtihel.louati@gmail.com (I.L.); hela.zouari@isbs.usf.tn (H.Z.-M.); 2 Department of Biology, Faculty of Science, University of Tabuk, Tabuk 47512, Saudi Arabia; alessiabdulrahman@gmail.com; 3 Department of Biology, Faculty of Science, Umm Al-Qura University, Makkah 24382, Saudi Arabia; aassaigh@uqu.edu.sa; 4 Institute of Biotechnology of Sfax, University of Sfax, BP 1175, Sfax 3038, Tunisia

**Keywords:** emerging pollutants, biodegradation, *Coriolopsis gallica*, β-lactam antibiotics, laccase

## Abstract

Due to widespread overuse, pharmaceutical compounds, such as antibiotics, are becoming increasingly prevalent in greater concentrations in aquatic ecosystems. In this study, we investigated the capacity of the white-rot fungus, *Coriolopsis gallica* (a high-laccase-producing fungus), to biodegrade ampicillin under different cultivation conditions. The biodegradation of the antibiotic was confirmed using high-performance liquid chromatography, and its antibacterial activity was evaluated using the bacterial growth inhibition agar well diffusion method, with *Escherichia coli* as an ampicillin-sensitive test strain. *C. gallica* successfully eliminated ampicillin (50 mg L^−1^) after 6 days of incubation in a liquid medium. The best results were achieved with a 9-day-old fungal culture, which treated a high concentration (500 mg L^−1^) of ampicillin within 3 days. This higher antibiotic removal rate was concomitant with the maximum laccase production in the culture supernatant. Meanwhile, four consecutive doses of 500 mg L^−1^ of ampicillin were removed by the same fungal culture within 24 days. After that, the fungus failed to remove the antibiotic. The measurement of the ligninolytic enzyme activity showed that *C. gallica* laccase might participate in the bioremediation of ampicillin.

## 1. Introduction

In recent years, greater attention has been paid to emerging pollutants, such as pesticides, drugs, and endocrine-disrupting chemicals (EDCs), in the aquatic environment [1,2,3]. Antibiotics are a group of pharmaceuticals that are widely used in both human [4] and veterinary medicine (particularly in farm animals and for the purpose of growth promotion) [5]. Their widespread use has necessitated the development of new antibiotics due to the proliferation of antibiotic-resistant pathogens [6]. A recent study reported an annual global antibiotic consumption of more than 200,000 tons [7], with the most common class, β-lactam antibiotics, constituting 50–70% of sales [8]. This increase in the consumption of antibiotics has been associated with an increase in their irrational use, from 28% to 65% [9]. In a 2015 study, despite its status as a low–middle-income country, Tunisia was found to have the highest antibiotic consumption rates, with 61% of consumers obtaining antibiotics directly from a pharmacist without a medical prescription [10].

Moreover, modern animal production practices are associated with the regular use of antimicrobials; global consumption of antimicrobials is estimated to rise from 63,151 ± 1560 tons to 105,596 ± 3605 tons between 2010 and 2030 (a 67% increase) [11]. The generation of pathogen-resistant antibiotics as a result of unrestricted use of antibiotics will have direct consequences for human health [12,13], promoting antibiotic resistance genes [14] and indirectly affecting the environment [15,16,17] via effluents from urban wastewater treatment plants (WWTPs) containing antibiotics and their residues, since they are not designed to eliminate them [18]. Therefore, an urgent and universal effort must be made to control the concentration of antibiotics and the development of multiple antibiotic-resistant bacteria [14].

Several studies have shown that wastewater from municipal conventional WWTPs could be a significant source of contamination of the aquatic environment by antibacterial agents [18]. Therefore, antibiotics have been detected in different environmental matrices, such as ground and surface water [19,20], soil and sediment samples [21,22], as well as in aquatic organisms [23]. They demonstrated pseudo-persistent behavior in the environment and their accumulation in different environmental compartments can threat human and animal existence and may have an impact on their health [24,25]. Indeed, using treated wastewater for irrigation may contaminate agricultural soils and lead to an uptake of residual antibiotics by plants [19,26]. As a result, the antibiotic residues that are present in the ecosystem provide an ideal setting for the acquisition and spread of antibiotic resistance genes, causing serious environmental problems [27].

Ampicillin, a semi-synthetic β-lactam, is part of a group of isoxazolyl penicillins (PI), which obtain their antimicrobial properties from the presence of a β-lactam ring [28]. Thus, their structures grant them resistance to degradation via conventional biological and chemical methods [29]. This antibiotic is widely used to treat infections in both human and veterinary medicine. Approximately 30% of ampicillin is excreted following oral administration, while 75% is excreted after intravenous use [30]. In an analysis of wastewater from a swine lagoon, the concentration of β-lactam antibiotics was approximately 2.1–3.5 μg L^−1^, which was close to the upper detection limit (2 μg L^−1^) [31]. Similarly, these antibiotics, such as amoxicillin, ampicillin, mezlocillin, flucloxacillin, and piperacillin, were detected at concentrations up to 48 ng L^−1^ in natural waters [32], while concentrations greater than 75.40 ng L^−1^ were observed in raw wastewater from the Sfax treatment plant [33]. As such, finding treatments that are capable of removing β-lactam antibiotics from water is an ongoing challenge [34].

Many researchers have attempted to remove antibiotics from aqueous solutions [35]. Processes such as Fenton reactions [36], UV/ZnO degradation [37,38], advanced oxidation [39,40,41], and adsorption [42,43] have been designed to degrade pharmaceutical waste in water matrices; however, biological methods are considered to be the optimal solution for antibiotic removal due to their eco-friendly nature [44]. In fact, most tested antibiotics are known to be biorecalcitrant under aerobic conditions [45], escaping from conventional wastewater treatment plants intact. In this light, non-biological methods, such as advanced oxidation processes, membrane separation, adsorption, coagulation, and combinations thereof, have been employed to remove antibiotics (and other pharmaceuticals) from water [25,39,41,46].

White-rot fungi (WRF) are a promising group of fungi that are known to be able to transform recalcitrant compounds [47]. They were applied to remove pharmaceutical residues from different environmental matrices [48]. These organisms have the potential to adsorb [49], transform, and even to mineralize [50] a large spectrum of xenobiotics due to a non-specific enzymatic system, including extracellular lignin-modifying enzymes (mainly laccase, lignin peroxidase, manganese peroxidase, and versatile peroxidase) and intracellular enzymes, such as the cytochrome P450 system, among others [51]. This oxidizing property could have useful applications in the removal of micropollutants, which are usually resistant to biodegradation, and seems to be a very promising approach to improving water effluent quality at WWTPs [52,53].

The aim of this study was to investigate the potential of the white-rot fungus, *Coriolopsis gallica*, to remove ampicillin under different operational conditions. This fungus was investigated due to the high-level production of ligninolytic enzymes, especially laccase, and the capacity to biodegrade recalcitrant pollutants, such as dyes, bisphenol, and fluoroquinolones [54,55,56]. Since the transformation of these molecules does not necessarily lead to a decrease in their activity, the residual antibacterial activity of the treated solutions was also investigated to ensure the efficiency of the treatment. To the best of our knowledge, this is the first study in which *C. gallica* has been used to degrade ampicillin.

## 2. Results

### 2.1. Degradation of AMP in Liquid Media

**Figure 1:**
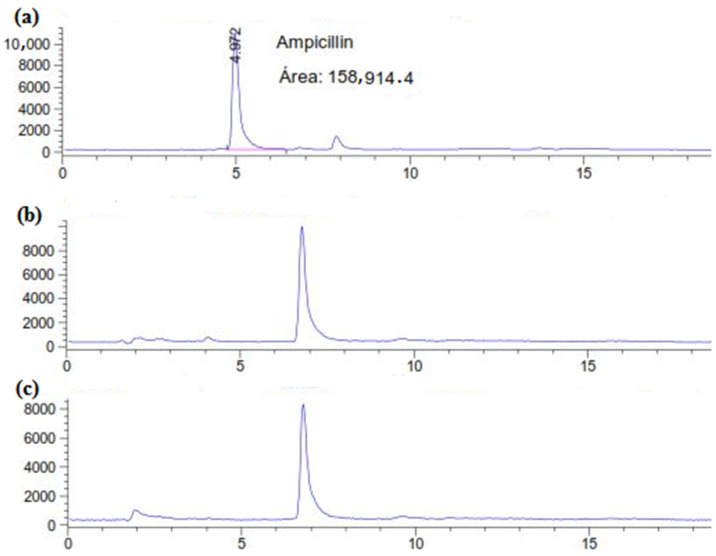
HPLC chromatograms of (**a**) the control (AMP at 50 mg L^−1^ in M7 medium) and the treated AMP after (**b**) 6 days and (**c**) 12 days.

The residual concentration of ampicillin in the supernatant was estimated directly using HPLC–UV analysis (270 nm) after 6 days and 12 days of treatment with *C. gallica* culture. According to the HPLC chromatograms (Figure 1), the untreated ampicillin (AMP) (control) was eluted from the column at 4.97 min. However, for the *C. gallica*-treated AMP, after 6 days and 12 days, the initial peak disappeared, and a new one was observed at 6.78 min (Figure 1b,c). These results indicate that the *C. gallica* strain successfully degraded the ampicillin during the 6-day incubation period.

### 2.2. Monitoring of Laccase and Antibacterial Activities at Different Conditions

#### 2.2.1. Effect of AMP Concentration

**Figure 2:**
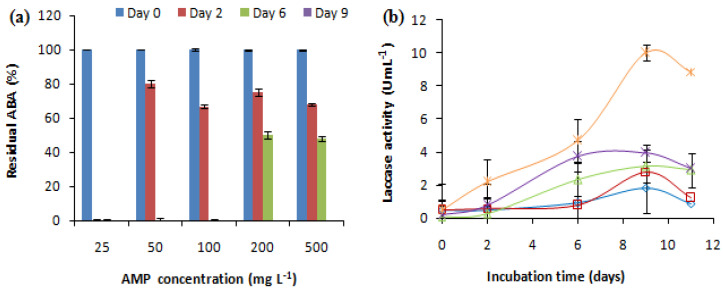
Theeffect of the initial antibiotic concentration on (**a**) the antibacterial activity removal and (**b**) the laccase activity concentration (0.15 mM Cu^2+^, 30 °C, 150 rpm). “♦”: 25 mg L^−1^; “■”: 50 mg L^−1^; “▲”: 100 mg L^−1^; “X”: 200 mg L^−1^; “*”: 500 mg L^−1^. Each experiment was performed in triplicate to obtain the final datapoints (mean ± standard deviation).

The effect of the initial antibiotic concentration on the removal of the antibacterial activity (ABA) was studied at AMP concentrations, ranging from 25 mg L^−1^ to 500 mg L^−1^. The *E. coli* growth inhibition zone related to ampicillin with or without fungal treatment (negative control) was measured. The changes in laccase activity during the treatment were also investigated (Figure 2). Non-treated ampicillin (abiotic control) maintained the same ABA during the experiment; however, the negative control (fungal culture without AMP) showed no activity against *E. coli*. Different concentrations of AMP showed variable effects on the antibacterial activity removal and laccase production. *C. gallica* eliminated the antibacterial activity at a wide range of AMP concentrations, ranging from 25 mg L^−1^ to 500 mg L^−1^ after 2, 6, and 9 days of treatment (Figure 2a). In fact, the ABA corresponding to 25 mg L^−1^ of AMP was successfully removed after 2 days of treatment in the presence of 0.28 U mL^−1^ of laccase, while the ABA corresponding to 50 mg L^−1^ and 100 mg L^−1^ of AMP was removed after 6 days of treatment in the presence of 0.8 and 2.33 U mL^−1^ of laccase, respectively. Meanwhile, the ABA corresponding to 200 mg L^−1^ and 500 mg L^−1^ of AMP was abated after 9 days of treatment in the presence of 3.94 U mL^−1^ and 10 U mL^−1^ of laccase, respectively (Figure 2b). As a result, the concentration of 500 mg L^−1^ of AMP was selected for use in the subsequent experiments.

#### 2.2.2. Effect of the Age of the Culture on AMP Removal

**Figure 3:**
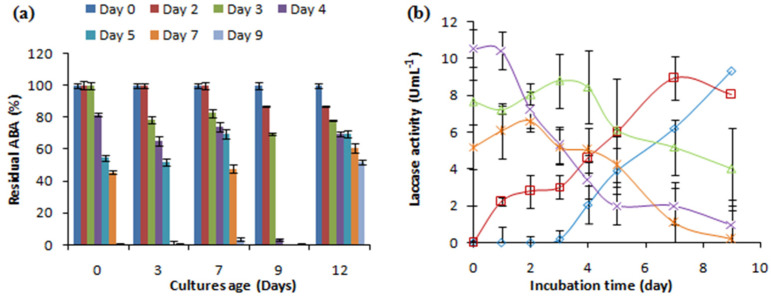
The effect of the age of the fungal culture on (**a**) the antibacterial activity removal and (**b**) the laccase activity concentration. “♦”: 0 days; “■”: 3 days; “▲”: 7 days; “X”: 9 days; “*”: 12 days. Each experiment was performed in triplicate to obtain the final datapoints (mean ± standard deviation).

To evaluate how the age of the culture affected the removal of the antibacterial activity of AMP, different fungal cultures aged 0, 3, 7, 9, and 12 days were supplemented with 500 mg L^−1^ of ampicillin and investigated with regard to laccase production and the removal of the antibacterial activity (Figure 3). The *C. gallica* cultures successfully eliminated the antibacterial activity of AMP (500 mg L^−1^), regardless of their age. The highest removal rate was achieved using a 9-day-old culture after 4 days of incubation with AMP, whereas a 12-day-old culture transformed only 50% of the initial ABA after 7 days of treatment (Figure 3a). Adding AMP re-stimulated laccase production regardless of the age of the culture (Figure 3b).

#### 2.2.3. Reusability of the Same Culture

**Figure 4:**
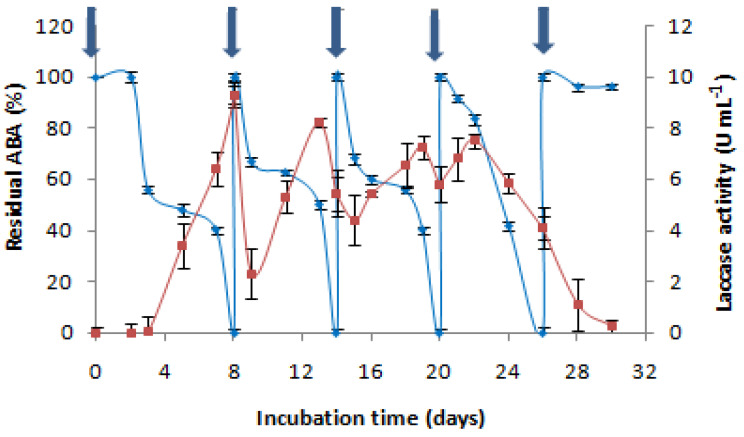
The effect of the cumulative addition of AMP (500 mg L^−1^) on the antibacterial activity removal “♦” and the laccase activity concentration “■”; 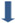: AMP addition to the culture medium. Each experiment was performed in triplicate to obtain the final datapoints (mean ± standard deviation).

To determine whether *C. gallica* (4-day-old culture) could be used for more than one cycle of treatment, consecutive additions of AMP were performed after the degradation of the first dose (500 mg L^−1^). The experiment was performed without nutrient supplementation. It was determined that four successive doses of 500 mg L^−1^ of AMP could be degraded by the same culture through 26 days of cultivation. Each time that AMP was added to the culture medium, laccase was induced, and the antibiotic was degraded. Then, laccase production decreased, rendering further degradation impossible. At that time, the medium exhibited a lack of nutrients, and the lysis of mycelia was observed. As such, we found that *C. gallica* efficiently eliminated AMP four successive times in the same culture (Figure 4).

## 3. Discussion

The objective of this experiment was to evaluate the potential of *C. gallica* to degrade a representative of the ß-lactam antibiotics that are currently in widespread use. This strain was already tested for its efficiency in degrading fluoroquinolones [56].We began by measuring the degradation of the ampicillin over time, comparing the chromatograms during the fungal treatment to ensure that the loss of the antibacterial activity of AMP was caused by a molecular transformation process of the antibiotic molecule. After 6 days of treatment, AMP was completely transformed. Compared with the results obtained in previous work, *C. gallica* showed greater efficiency in terms of the initial antibiotic concentration, the percentage of degradation, and the rate of molecular transformation. Many researchers have described how β-lactam antibiotics are transformed by ligninolytic fungi and their enzymes. Lucas et al. (2016) reported that 96% of β-lactam antibiotics (initial concentration 10 µg L^−1^) were eliminated by *Trametes versicolor* after 15 days of treatment [57], while Copete-Pertuz et al. (2018) reported a 100% removal of oxacillin (16 mg L^−1^), cloxacillin (17.5 mg L^−1^), and dicloxacillin (19 mg L^−1^) by *Leptosphaerulina* sp. after 6, 7, and 8 days of treatment, respectively, under the action of laccase and versatile peroxidase [58].

We evaluated the ability of *C. gallica* to remove the antibacterial activity of AMP under different culture conditions, measuring the laccase activity in each condition. Few researchers have explored the correlation between antibiotic biotransformation and laccase production in depth, but a recent study reported the involvement of extracellular enzymes in antibiotic degradation, with a putative major role for laccases [56]. In this work, we focused on laccase, as it could be easily repurposed as free or grafted systems to support sustainable processes. The removal rate of AMP was affected by the initial antibiotic concentration, showing variable effects based on the antibacterial activity removal and the laccase production. The presence of AMP in the culture media was observed to induce laccase production and increasing the concentration of AMP led to an increase in the laccase activity; however, high initial concentrations of AMP required a longer treatment duration. These results are in agreement with those reported by Dhawan et al. (2015), who found that nine different antibiotics affected fungal growth, protein release, and laccase production in *Cyathus bulleri* (5.3 U mL^−1^) and *Pycnoporus cinnabarinus* (10.9 U mL^−1^) to different extents. Interestingly, apramycin sulfate (500 mg L^−1^) stimulated the maximum laccase production (23.3 U mL^−1^) in *P. cinnabarinus*. However, ampicillin trihydrate (200 mg L^−1^) induced laccase production in *C. bulleri* from 5.5 U mL^−1^ to 10.6 U mL^−1^ [59]. Praveen and Reddy (2012) also described the role of nine antibiotics in laccase induction in *Stereum ostrea* (27.48 U mL^−1^) and *Phanerochaete chrysosporium* (1.3 U mL^−1^). In their study, tetracycline (500 mg L^−1^) stimulated the maximum laccase production (33.4 U mL^−1^ and 4 U mL^−1^) and ampicillin (200 mg L^−1^) increased laccase production (27.48 U mL^−1^ and 1.742 U mL^−1^) in *S. ostrea* and *P. chrysosporium*, respectively [60].

Next, we investigated the effect of the age of the culture on the removal of the antibacterial activity of AMP. The addition of AMP (500 mg L^−1^) to different *C. gallica* cultures affected the kinetics of both the laccase production and the ABA removal. In the absence of AMP and under the same cultivation conditions (M7, 30 °C, 150 rpm, + Cu^2+^), *C. gallica* produced the maximum laccase quantity on day 9, after which production declined [61]. In this study, the addition of AMP re-stimulated the laccase production regardless of the age of the culture. A possible explanation for this finding is that the fungi may have mistaken AMP for a phenolic substrate. Similarly, Sandhu and Arora (1985) observed the induction of laccase production in *Polyporus sanguineus* in the presence of different phenolic compounds. Furthermore, they proposed that white-rot fungi may sense the antibiotic as a phenolic substrate that must be attacked and detoxified by means of enzymatic transformation [62]. *Phlebia radiata* has also been found to produce lignin-modifying enzymes for detoxification purposes in response to the presence of toxic compounds in its environment [63].

The reusability of a treatment process is a crucial factor in industrial applications, representing a high priority for manufacturers due to economic considerations. Four successive doses of AMP (500 mg L^−1^) were degraded by the same *C. gallica* culture in this study. Laccase production was re-stimulated each time AMP was added to the same culture; therefore, it could be concluded that, even if laccase is not the key enzyme responsible for AMP degradation, it may be involved in its biotransformation reaction. Yang et al. (2017) investigated ampicillin degradation by immobilized *Cerrena* laccase. In the absence of a redox mediator, the degradation efficiency was <40%, whereas adding the mediator, ABTS, increased the degradation efficiency to 55% [64]. Furthermore, Zhang et al. (2020) reported the effective degradation of ampicillin (100%) by free and immobilized laccase in water and proposed two degradation pathways involved in the oxidation of ampicillin by laccase [65]. In the first degradation pathway, the process began with the oxidation of the sulfur atom of ampicillin, which resulted in the generation of a sulfur–oxygen bond in the so-called molecules (TP365 and TP397). In the second pathway, however, the β-lactam ring of ampicillin was directly opened and oxidized to generate TP366; therefore, we could attribute the loss of ampicillin activity to the cleavage of the β-lactam ring by laccase.

These works illustrate the efficiency of the fungal remediation process over the large range of ß-lactam found in the environment, and the gap to be filled in order to understand how the fungi manage with these pollutants and how they can be used to support sustainable processes. In conclusion, several enzymes, including laccases, could be the target of future research to evaluate purified enzymes and identify the main by-products. It would be better to evaluate free and immobilized enzymes alone or in the presence of a chemical mediator, such as HBT, to improve the current degradation performances.

## 4. Materials and Methods

### 4.1. Chemicals and Reagents

Ampicillin sodium salt (CAS No. 69-52-3, ≥98.0%) and 2,6-dimethoxyphenol (2,6-DMP, 99%) were obtained from SigmaAldrich (Burlington, MA, USA). All other chemicals and solvents used in this study were of an HPLC or reagent grade.

The different concentrations of AMP, ranging from 25 mg L^−1^ to 500 mg L^−1^, were obtained usingappropriate dilution of the stock solution in distilled water. The maximum absorbance (λmax) of AMP was determined using UV–visible spectrophotometry (JENWAY 7315 Spectrophotometer, Cole-Parmer, Inc., Vernon Hills, IL, USA). The chemical structure and some characteristics of AMP are shown in Table 1.

### 4.2. Microorganisms

The fungal strain used in this study was *C. gallica* CLBE55, a white-rot fungus isolated from a Tunisian forest biotope in the northwest in 2008 (GPS coordinates: 36.653681, 8.904576) and maintained using subculturing on 2% malt extract agar slants at pH 5 and 30 °C at 30-day intervals [66].

*Escherichia coli* (ATCC 25922) was used as the test strain with which to measure the residual antibacterial activity of the treated solutions.

### 4.3. Experimental Procedures

#### 4.3.1. Follow-Up of AMP Concentration Time-Course in the Culture Medium

The experiments were performed in 500 mL Erlenmeyer flasks containing 150 mL of the M7 medium and inoculated with 1% of homogenized mycelium. The M7 medium contained (per liter): glucose, 10 g; peptone, 5 g; yeast extract, 1 g; ammonium tartrate, 2 g; KH_2_PO_4_, 1 g; MgSO_4_·7H_2_O, 0.5 g; KCl, 0.5 g; and trace element solution, 1 mL. The composition of the trace element solution per liter was as follows: B_4_O_7_Na_2_·10H_2_O, 0.1 g; CuSO_4_·5H_2_O, 0.01 g; FeSO_4_·7H_2_O, 0.05 g; MnSO_4_·7H_2_O, 0.01 g; ZnSO_4_·7H_2_O, 0.07 g; (NH_4_)6Mo_7_O_24_·4H_2_O, 0.01 g. The pH of the solution was adjusted to 5.5. The cultures were incubated at 30 °C on a rotary shaker (160 rpm). M7 was supplemented with CuSO_4_·5H_2_O (150 µM) after 3 days of cultivation to induce laccase [61]. Each experiment was conducted in triplicate and included non-inoculated controls containing 150 mL of the same medium. After 4 days of cultivation, ampicillin was added to the flasks to produce the desired concentration from a stock solution in water. The flasks were incubated in the dark, on an orbital shaker in the same conditions mentioned previously in order to exclude the influence of light on the ampicillin stability. In the time-course experiments, 1.5 mL samples were periodically withdrawn, filtered, and analyzed to assess the laccase and antibacterial activity. The samples were maintained at a temperature of −20 °C until the HPLC analysis.

#### 4.3.2. In Vitro Analysis of Residual AMP

##### Effect of the Antibiotic Concentration

To study the ability of *C. gallica* to eliminate the antibacterial activity at high concentrations, different doses of ampicillin were added to the culture medium at a final concentration ranging between 25 mg L^−1^ and 500 mg L^−1^ on the fourth day of cultivation. The samples were withdrawn periodically, centrifuged, and analyzed to determine the residual antibacterial and laccase activity.

##### Effect of the Age of the Mycelia

To investigate the influence of the age of the mycelia on the removal of the antibacterial activity of ampicillin, the antibiotic solution was added to cultures at different stages of fungal growth and varying laccase production levels. The final concentration of ampicillin was 500 mg L^−1^, and the ages of the tested cultures were 0, 3, 7, 9, and 12 days.

##### Effect of the Consecutive Addition of Ampicillin

To study the potential of a *C. gallica* culture for the consecutive treatment of ampicillin, the antibiotic was re-added to the same culture at the same concentration (500 mg L^−1^) once the antibacterial activity of the previous concentration decreased to an undetectable level.

### 4.4. Analytical Procedures

#### 4.4.1. HPLC Analysis of Ampicillin in *C. gallica* Culture Filtrate

The concentration of AMP in the tested culture was measured using HPLC–UV (Agilent 1100 Series, Agilent Technologies, Waldbronn, Germany) equipped with a micro-vacuum degasser (Agilent 1100 Series, Agilent Technologies, Waldbronn, Germany), quaternary pump, diode array, and mass detector (Agilent Technologies 61120 Quadrupole LC/MS, Agilent Technologies, Waldbronn, Germany) at a wavelength of 270 nm. The separation was performed using a ZORBAX SB-C18 (150 mm × 4.6 mm, 5 µm) column. The mobile phase was a mixture of A (H_2_O + 0.1% formic acid) and B (acetonitrile + 0.1% formic acid) [64] at a flow rate of 1 mL min^−1^ (initial, 10% B; 15 min, 90% B; 25 min, 90% B; 26 min, 10% B; 36 min, 10% B). The column temperature was 35 °C and 10 µL of each sample was injected.

#### 4.4.2. Antibacterial Activity Assay

The antibacterial activity (ABA) of ampicillin before and after treatment was also evaluated using the agar well diffusion method [67]. *E. coli* cells were cultured overnight at 37 °C with shaking (150 rpm) in a lysogeny broth (LB) medium. Petri dishes containing an LB agar medium were inoculated aseptically with a suspension of 106 cells per mL from the young culture. After drying, the agar was perforated with the upper part of a Pasteur pipette. The resulting cavities were filled with samples obtained at different treatment times (50 µL per well).An abiotic control (AMP in culture medium without fungus) and a negative control (fungal culture without AMP) were evaluated in parallel with the tests described above. The experiments were performed in triplicate. The Petri dishes were incubated at 37 °C for 24 h. The growth inhibition was calculated by measuring the diameter of the growth inhibition against the control as follows:

$$\mathrm{Removal}\;\mathrm{efficiency}\left(\%\right)=\frac{\left(D_{0}-D_{t}\right)}{D_{0}}*100$$

where D_0_ and D_t_ are the diameters of the growth inhibition zone (mm) corresponding to AMP added to the culture on day 4 and the residual AMP at culture time, t, respectively.

#### 4.4.3. Laccase Activity Assay

As oxidative enzymes can potentially participate in antibiotic degradation, laccase activity was assayed during the culture period by monitoring the oxidation of 10 mM of 2,6-dimethoxyphenol (DMP) (469 nm, ε_469_ = 27,500 M^−1^cm^−1^) in a reaction mixture solution containing 100 mM of citrate buffer at pH 5. One unit of enzyme activity was defined as the amount of enzyme oxidizing 1 µM of substrate min^−1^ [68].

## 5. Conclusions

This study investigated the degradation of ampicillin by *C. gallica* under different culture conditions. The selected fungus successfully degraded AMP in a liquid medium after 6 days of treatment. Based on the activity assays, *C. gallica* laccase was involved in the enzymatic degradation of ampicillin and contributed to the removal of its antibacterial activity. The loss of antibacterial activity could be attributed to the cleavage of the β-lactam ring in response to laccase. Further experiments, such as proteomic analysis, should be performed to investigate other enzymes that may be involved in ampicillin degradation and identify the degradation products generated during the treatment process.

The strong performance of *C. gallica* in the effective removal of ampicillin makes it a promising candidate for environmental recovery, with potential applications in eco-friendly biological treatment processes to remove antibiotics from wastewater; a single culture of fungus could be deployed for prolonged use at a wastewater plant without the need to replenish the nutrients.

## Figures and Tables

**Table 1:** Physicochemical characteristics of AMP.

Antibiotic	Class	λmax (nm)	Chemical Structure
Ampicillin	β-lactam	204	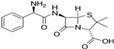

## Data Availability

Data will be available on request from corresponding author.
